# Tertiary sulphonamide derivatives as dual acting small molecules that inhibit LSD1 and suppress tubulin polymerisation against liver cancer

**DOI:** 10.1080/14756366.2021.1917564

**Published:** 2021-07-19

**Authors:** Lijuan Ding, Feng Wei, Nanya Wang, Yue Sun, Qiang Wang, Xia Fan, Ling Qi, Shudong Wang

**Affiliations:** a The First Hospital of Jilin University, Changchun, China; b The Sixth Affiliated Hospital of Guangzhou Medical University, Qingyuan People’s Hospital, Qingyuan, China

**Keywords:** Tertiary sulphonamide, liver cancer, Bel-7402 cells, tubulin polymerisation, LSD1

## Abstract

A series of tertiary sulphonamide derivatives were synthesised and evaluated for their antiproliferative activity against liver cancer cell lines (SNU-475, HepG-2, and Bel-7402). Among these tertiary sulphonamides, compound **17a** displayed the best anti-liver cancer activity against Bel-7402 cells with an IC_50_ value of 0.32 μM. Compound **17a** could effectively inhibit tubulin polymerisation with an IC_50_ value of 1.27 μM. Meanwhile, it selectively suppressed LSD1 with an IC_50_ value of 63 nM. It also concentration-dependently inhibited migration against Bel-7402 cells. Importantly, tertiary sulphonamide **17a** exhibited the potent antitumor activity *in vivo*. All these findings revealed that compound **17a** might be a tertiary sulphonamide-based dual inhibitor of tubulin polymerisation and LSD1 to treat liver cancer.

## 1. Introduction

**Figure 1.**
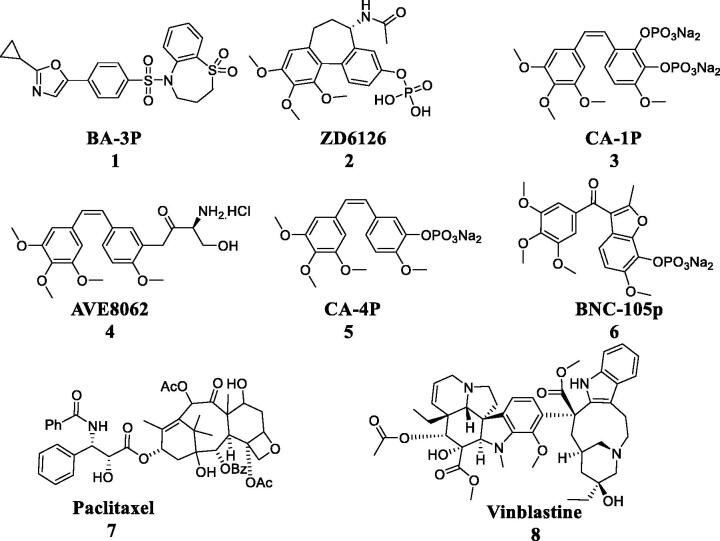
Microtubule-targeting agents.

Microtubules as ubiquitous fibrous structures in eukaryotic cells are the key component of cytoskeleton^1^. They are spherical proteins composed of α- and β-tubulin heterodimers, which display significant functions in the formation and maintenance of cell shape, intracellular transport, cell division and organellar movement^2^. Benzenesulphonamide derivative **BA-3P** was synthesised as a anti-prostate cancer agent targeting tubulin^2^. Furthermore, several microtubule-binding drugs (**ZD6126**, **CA-1P**, **AVE8062**, **CA-4P**, **BNC-105p**, **Paclitaxel** and **Vinblastine**) have been successfully employed in clinical use (Figure 1)^3^. These microtubule-targeting agents have the great potential to be anticancer drugs, which could affect the process of microtubule homoeostasis, induce tumour cell apoptosis and arrest cell cycle^4^. Hence, the development of microtubule-binding agents is a hot field in the research of novel antitumor drugs.

**Figure 2.**
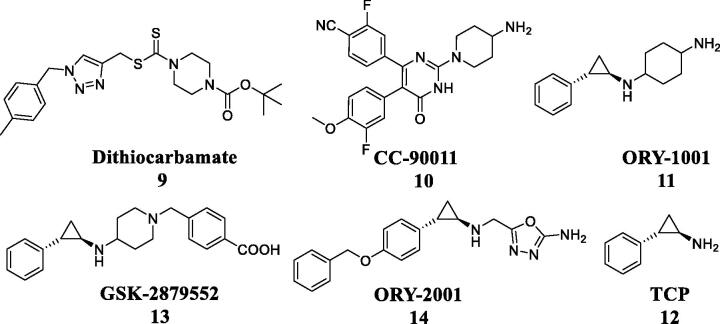
Structures of LSD1 inhibitors.

In 2004, the first histone demethylase (Lysine Specific Demethylase 1, LSD1) was discovered, which was a flavin-dependent monoamine oxidase protein containing 852 amino acids with a molecular weight of 110 kDa^5^. LSD1 could specifically induce the demethylation reaction of the methylated H3K4 and H3K9 sites and also display various roles in the development of human cancer, including epithelial mesenchymal transition, cell proliferation, cell cycle arrest, chromosome segregation, and cell migration^6^. LSD1 as a new anti-tumour target is abnormally expressed in gastric cancer, liver cancer, breast cancer, oesophageal cancer and acute myeloid leukaemia^7^. **Dithiocarbamate 9** as a LSD1 inhibitor potently inhibited the growth of LSD1 overexpressing gastric tumour *in vivo* ^8^. In addition, several LSD1 inhibitors (**CC-90011**, **ORY-1001**, **GSK-2879552**, **ORY-2001** and **TCP**) presently undergo clinical assessment for cancer therapy (Figure 2)^8^^,^^9^.

**Figure 3.**
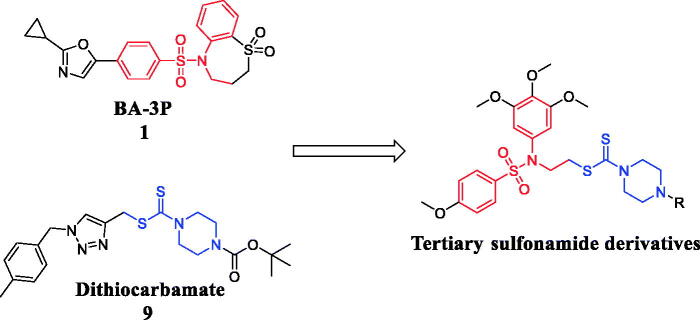
Discovery of dual acting small molecules.

As shown in Figure 3, the design strategy based on structures of **BA-3P** and **Dithiocarbamate** yielded a novel scaffold which has three parts: (i) a tertiary sulphonamide moiety from **BA-3P** as the microtubule-binding fragment, (ii) a dithiocarbamate moiety from compound **9** as the potential unit targeting LSD1, (iii) different substituents attaching the piperazine ring to investigate their molecular diversity. Following these success in the development of microtubule-targeting agents and LSD1 inhibitors, we also describe the synthesis and preliminary anticancer mechanisms of these tertiary sulphonamide analogues as dual acting small molecules that inhibit LSD1 and suppress tubulin polymerisation in this work. Biological experiments of enzymatic and cellular levels were performed to investigate their binding effects targeting LSD1 and tubulin. The antitumor effects of novel tertiary sulphonamide derivatives were also explored against liver cancer.

## 2. Materials and methods

### 2.1. Chemistry

All chemical reagents and solvents in this work were purchased (Aladdin, Shanghai, China) or Innochem Co. Ltd. (Beijing, China). The melting points of all tertiary sulphonamide analogues were measured using micromelting point tester (Hebi Huatai Electronics Co., Ltd, Henan, China). ^1^H-NMR and ^13 ^C-NMR spectra of tertiary sulphonamide analogues were determined on a nuclear magnetic resonance apparatus (WNMR-I, BRUKER, Beijing, China). DMSO-*d*_6_ and CDCl3 were used as nuclear magnetic solvents. HRMS were obtained on a mass spectrometer (MALDI-2, BRUKER, Beijing, China).

### 2.2. General procedure for the synthesis of intermediates 15 and 16

To a solution of 4-methoxybenzenesulfonyl chloride (5 mmol) in dichloromethane (25 ml) was added 3,4,5-trimethoxyaniline (5.5 mmol) and KOH (5 mmol). The reaction mixture was stirred for 5 h at room temperature. The system was purified with column chromatography (hexane: EtOAc = 9:1) to obtain the intermediate **15**. To a solution of intermediate **15** (3 mmol) in acetone (15 ml) was added 1,2-dibromoethane (3.6 mmol) and potassium carbonate (3 mmol). The mixture was stirred at 80 °C. Upon completion, the system was evaporated to give the crude products. The residues were purified with column chromatography (hexane: EtOAc = 9:1) to obtain the intermediate **16**.

#### 2.2.1. 4-Methoxy-N-(3,4,5-trimethoxyphenyl)benzenesulfonamide (15)

Yield: 84%, white solid, m.p.:1 1 4 ∼ 116 °C. ^1^H NMR (400 MHz, DMSO-*d*_6)_ δ 9.97 (s, 1H), 7.78 − 7.65 (*m*, 2H), 7.16 − 7.00 (*m*, 2H), 6.38 (*s*, 2H), 3.80 (*s*, 3H), 3.65 (*s*, 6H), 3.56 (*s*, 3H). ^13 ^C NMR (100 MHz, DMSO-*d*_6_) δ 162.43, 152.91, 133.99, 133.84, 131.03, 129.01, 114.32, 97.72, 60.01, 55.70, 55.59. HRMS (*m*/*z*) calcd. C_16_H_20_NO_6_S, [M + H] ^+^ *m*/*z*: 354.1016, found: 354.1011.

#### 2.2.2. N-(2-bromoethyl)-4-methoxy-N-(3,4,5-trimethoxyphenyl)benzenesulfonamide (16)

Yield: 90%, white solid, m.p.:1 1 6 ∼ 118 °C. ^1^H NMR (400 MHz, CDCl_3_) δ 7.60 − 7.43 (*m*, 2H), 6.95 − 6.80 (*m*, 2H), 6.18 (*s*, 2H), 3.78 (dd, *J* = 12.2, 6.5 Hz, 8H), 3.65 (*s*, 6H), 3.34 (*t*, *J* = 7.4 Hz, 2H). ^13 ^C NMR (100 MHz, CDCl_3_) δ 162.21, 152.27, 137.24, 133.46, 129.03, 128.75, 112.96, 105.60, 59.90, 55.18, 54.67, 51.75, 27.96. HRMS (*m*/*z*) calcd. C_18_H_23_BrNO_6_S, [M + H] ^+^ *m*/*z*: 460.0434, found: 460.0429.

### 2.3. General procedure for the synthesis of compounds 17a∼17f

The intermediate **16** (1 mmol), different piperazine derivatives (1.5 mmol), Na_3_PO_4_.12H_2_O (0.1 mmol) and CS_2_ (3 mmol) were added to acetone (10 ml). The mixture was stirred at 0 °C for 3 h. Then NaN3 (1.7 g) was added to the mixture and the system was stirred at room temperature for 1 h. Upon completion, the reaction mixture was concentrated under vacuum and the residue was purified with column chromatography (hexane: EtOAc = 10:1) to obtain compounds **17a∼17f**.

#### 2.3.1. 2-((4-Methoxy-N-(3,4,5-trimethoxyphenyl)phenyl)sulfonamido)ethyl-4-methylpiperazine-1-carbodithioate (17a)

Yield: 67%, white solid, m.p.: 142 ∼ 144 °C. ^1^H NMR (400 MHz, CDCl_3_) δ 7.51 (d, *J* = 8.8 Hz, 2H), 6.86 (d, *J* = 8.9 Hz, 2H), 6.24 (*s*, 2H), 4.23 (*s*, 2H), 3.85 (*s*, 2H), 3.78 (d, *J* = 3.5 Hz, 6H), 3.76 − 3.70 (*m*, 2H), 3.67 (*s*, 6H), 3.46 − 3.23 (*m*, 2H), 2.50 − 2.31 (*m*, 4H), 2.25 (*s*, 3H). ^13 ^C NMR (100 MHz, CDCl_3_) δ 194.81, 162.05, 152.03, 136.91, 133.38, 129.01, 128.68, 112.88, 105.42, 59.91, 55.18, 54.64, 53.33, 48.62, 44.58, 33.99. HRMS (*m*/*z*) calcd. C_24_H_34_N_3_O_6_S_3_, [M + H] ^+^ *m*/*z*: 556.1616, found: 556.1610.

#### 2.3.2. 2-((4-Methoxy-N-(3,4,5-trimethoxyphenyl)phenyl)sulfonamido)ethyl-4-ethylpiperazine-1-carbodithioate (17b)

Yield: 86%, white solid, m.p.: 150 ∼ 152 °C. ^1^H NMR (400 MHz, CDCl_3_) δ 7.51 (d, *J* = 8.9 Hz, 2H), 6.97 − 6.75 (*m*, 2H), 6.24 (*s*, 2H), 4.23 (*s*, 2H), 3.85 (*s*, 2H), 3.78 (d, *J* = 3.5 Hz, 6H), 3.75 − 3.70 (*m*, 2H), 3.67 (*s*, 6H), 3.43 − 3.28 (*m*, 2H), 2.57 − 2.25 (*m*, 6H), 1.03 (*t*, *J* = 7.2 Hz, 3H). ^13 ^C NMR (100 MHz, CDCl_3_) δ 194.59, 162.04, 152.02, 136.90, 133.37, 129.01, 128.68, 112.88, 105.41, 59.91, 55.18, 54.63, 51.10, 50.85, 48.63, 33.95, 10.95. HRMS (*m*/*z*) calcd. C_25_H_36_N_3_O_6_S_3_, [M + H] ^+^ *m*/*z*: 570.1769, found: 570.1766.

#### 2.3.3. 2-((4-Methoxy-N-(3,4,5-trimethoxyphenyl)phenyl)sulfonamido)ethyl-4–(2-hydroxyethyl)piperazine-1-carbodithioate (17c)

Yield: 90%, white solid, m.p.: 146 ∼ 148 °C. ^1^H NMR (400 MHz, DMSO-*d*_6_) δ 7.57 (d, *J* = 8.8 Hz, 2H), 7.12 (d, *J* = 8.9 Hz, 2H), 6.29 (*s*, 2H), 4.47 (*t*, *J* = 5.3 Hz, 1H), 4.18 (*s*, 2H), 3.84 (*s*, 5H), 3.75 (*t*, *J* = 6.8 Hz, 2H), 3.66 (*s*, 3H), 3.64 (*s*, 6H), 3.51 (*q*, *J* = 5.9 Hz, 2H), 3.29 (*t*, *J* = 6.9 Hz, 2H), 2.48 (d, *J* = 5.0 Hz, 4H), 2.42 (*s*, 2H). ^13 ^C NMR (100 MHz, DMSO-*d*_6_) δ 194.00, 162.79, 152.57, 137.25, 134.06, 129.79, 129.15, 114.31, 106.49, 60.06, 59.44, 58.48, 55.87, 55.74, 52.47, 49.07, 34.55. HRMS (*m/z*) calcd. C_25_H_36_N_3_O_7_S_3_, [M + H] ^+^ *m*/*z*: 586.1719, found: 586.1715.

#### 2.3.4. 2-((4-Methoxy-N-(3,4,5-trimethoxyphenyl)phenyl)sulfonamido)ethyl-4-acetylpiperazine-1-carbodithioate (17d)

Yield: 90%, white solid, m.p.: 152 ∼ 154 °C. ^1^H NMR (400 MHz, CDCl_3_) δ 7.50 (d, *J* = 8.9 Hz, 2H), 6.86 (d, *J* = 8.9 Hz, 2H), 6.22 (*s*, 2H), 3.99 (dd, *J* = 70.5, 66.9 Hz, 4H), 3.78 (d, *J* = 3.6 Hz, 6H), 3.73 (*t*, *J* = 7.0 Hz, 2H), 3.66 (d, *J* = 8.5 Hz, 8H), 3.57 − 3.47 (*m*, 2H), 3.37 (*t*, *J* = 7.0 Hz, 2H), 2.06 (*s*, 3H). ^13 ^C NMR (100 MHz, CDCl_3_) δ 168.32, 162.09, 152.09, 136.98, 133.35, 128.99, 128.67, 112.91, 105.45, 59.92, 55.20, 54.66, 48.50, 44.14, 39.54, 34.12, 20.33. HRMS (*m*/*z*) calcd. C_25_H_34_N_3_O_7_S_3_, [M + H] ^+^ *m*/*z*: 584.1565, found: 584.1559.

#### 2.3.5. 2-((4-Methoxy-N-(3,4,5-trimethoxyphenyl)phenyl)sulfonamido)ethyl-4–(4-fluorophenyl)piperazine-1-carbodithioate (17e)

Yield: 72%, white solid, m.p.: 166 ∼ 168 °C. ^1^H NMR (400 MHz, CDCl_3_) δ 7.67 − 7.37 (*m*, 2H), 7.06 − 6.63 (*m*, 6H), 6.24 (*s*, 2H), 4.36 (*s*, 2H), 4.02 (*s*, 2H), 3.78 (*s*, 6H), 3.77 − 3.71 (*m*, 2H), 3.67 (*s*, 6H), 3.45 − 3.30 (*m*, 2H), 3.22 − 3.00 (*m*, 4H). ^13 ^C NMR (100 MHz, CDCl_3_) δ 195.14, 162.07, 152.08, 145.95, 133.38, 129.01, 117.51, 117.46, 117.38, 114.92, 114.69, 112.90, 105.50, 59.92, 55.21, 54.64, 48.86, 48.62, 34.08. HRMS (*m*/*z*) calcd. C_29_H_35_FN_3_O_6_S_3_, [M + H] ^+^ *m*/*z*: 636.1678, found: 636.1672.

#### 2.3.6. Tert-butyl-4-(((2-((4-methoxy-N-(3,4,5-trimethoxyphenyl)phenyl)sulfonamido)ethyl)thio)carbonothioyl)piperazine-1-carboxylate (17f)

Yield: 52%, white solid, m.p.: 162 ∼ 164 °C. ^1^H NMR (400 MHz, CDCl_3_) δ 7.51 (d, *J* = 8.9 Hz, 2H), 6.86 (d, *J* = 8.9 Hz, 2H), 6.23 (*s*, 2H), 4.34 − 4.02 (*m*, 2H), 3.89 (dd, *J* = 54.5, 9.9 Hz, 2H), 3.78 (d, *J* = 3.6 Hz, 6H), 3.76 − 3.70 (*m*, 2H), 3.67 (*s*, 6H), 3.51 − 3.41 (*m*, 4H), 3.41 − 3.32 (*m*, 2H), 1.40 (*s*, 9H). ^13 ^C NMR (100 MHz, CDCl_3_) δ 195.42, 162.08, 153.42, 152.08, 137.01, 133.37, 129.01, 128.74, 112.90, 105.49, 79.64, 59.91, 55.21, 54.64, 48.57, 34.06, 27.35. HRMS (*m*/*z*) calcd. C_28_H_40_N_3_O_8_S_3_, [M + H] ^+^ *m*/*z*: 642.1985, found: 642.1978.

### 2.4. Cell culture and MTT assay

Cell lines were purchased from Servicebio (Wuhan, China) and cultured in RPMI1640 (Servicebio, Wuhan, China) with 10% foetal bovine serum and 1% antibiotic agent (Servicebio, Wuhan, China) at 37 °C. With the treatment of synthetic compounds, cells were cultured for 48 h and added with MTT solution (Servicebio, Wuhan, China). After 4 h, medium was removed and DMSO was added to culture for 30 min. Finally, the absorbance value was obtained by a microplate reader (Multiskan Sky, Thermo Fisher, Shanghai, China).

### 2.5. The inhibitory activity of tubulin polymerisation *in vitro*

Firstly, 1.05 mmol/L ethylenebis(oxyethylenenitrilo)tetraacetic acid, 84 mmol/L piperazine-1,4-bisethanesulfonic acid, 0.525 mmol/L magnesium chloride, 10.71% (v/v) glycerol and 1 mmol/L adenosine triphosphate (Servicebio, Wuhan, China) were added in a tube to prepare the buffer solution. Then, tubulin was resuspended in the buffer solution with the treatment of tertiary sulphonamide derivatives at different concentrations. The reaction system was measured by a spectrophotometer (Multiskan Sky, Thermo Fisher, Shanghai, China) to obtain the fluorescence intensity.

### 2.6. Immunostaining assay

Liver cancer Bel-7402 cells were cultured on the slices for 24 h and treated with tertiary sulphonamide derivatives for 48 h. Then, Bel-7402 cells were fixed by 4% paraformaldehyde solution (Shanghai Yuanye Bio-Technology Co., Ltd, Shanghai, China) for 30 min. Bovine albumin was used to block the system for 30 min. Bel-7402 cells were cultured with β-tubulin and incubated for 24 h at 4 °C. Finally, 4′,6-diamidino-2-phenylindole (DAPI) was added in a dark room to obtain images of immunostaining (Confocal laser scanning microscope, FV3000RS, Beijing, China).

### 2.7. The inhibitory activity of LSD1 in vitro

Tertiary sulphonamide derivatives at different concentrations (0.025 μM, 0.05 μM, 0.1 μM, 0.15 μM and 0.2 μM) were incubated with the recombinant of cDNA encoding LSD1 (Servicebio, Wuhan, China). Then, the fluorescence values of tertiary sulphonamide derivatives were recorded (excitation wavelength: 530 nm, emission wavelength: 590 nm) to investigate their inhibition rates against LSD1 (Multiskan Sky, Thermo Fisher, Shanghai, China).

### 2.8. Western blot

Liver cancer Bel-7402 cells were cultured for 24 h and treated with tertiary sulphonamide derivative **17a** for 48 h at 37 °C. Protein lysates were harvested from the treated Bel-7402 cells. Nitrocellulose membrane was used to perform polyacrylamide gelelectrophoresis. H3K9me1 and H3K9me2 (Servicebio, Wuhan, China) were treated with the nitrocellulose membrane at 4 °C for 12 h. GAPDH as the secondary antibody was incubated for 1 h at room temperature (Shanghai Yuanye Bio-Technology Co., Ltd, Shanghai, China).

### 2.9. Migration assay

Liver cancer Bel-7402 cells were cultured in the upper chambers of a Transwell 24-well plate (Servicebio, Wuhan, China) for 24 h. 1% FBS and 20% FBS were added into the upper chambers and lower chambers respectively. Bel-7402 cells were treated with tertiary sulphonamide derivative **17a** for 48 h. Then, all medium was removed and Bel-7402 cells were fixed with ethanol for 30 min. Finally, Bel-7402 cells from the upper chambers were stained with haematoxylin solution (Servicebio, Wuhan, China) for 30 min.

### 2.10. Docking analysis

Binding modes of tertiary sulphonamide derivative **17a** were analysed using the autodock software (Scripps Research Institute, California, America). PDB codes of tubulin and LSD1 were 1SA0 and 2H94 respectively. Tertiary sulphonamide derivative **17a** and targeted proteins were converted to pdbqt formats by Open Babel. AutoGrid and AutoDock were performed to obtain the appropriate binding conformations. Hydrogen bonds and hydrophobic effects of tertiary sulphonamide derivative **17a** targeting LSD1 and tubulin were investigated from the docking results. Finally, the docking procedures were verified and analysed again based on these docking results.

### 2.11. Xenograft study and haematoxylin and eosin staining

Xenograft models using Bel-7402 cells was established in nude mice (Jiangsu ALF Biotechnology Co., LTD, Jiangsu, China). Then, mice-bearing tumours were divided into two groups (vehicle and 60 mg/kg group). Nude mice were treated with tertiary sulphonamide derivative **17a** by the intragastric administration for 21 days. Tumour volume and tumour weight of treated mice were measured. Haematoxylin & eosin staining experiments of heart, liver, spleen, lung and kidney were performed according to reported references^10^^,^^11^. We confirm that the animal study has received approval from the ethics committee of The First Hospital of Jilin University (Experimental number: DLJ-2020–05).

## 3. Results and discussion

### 3.1. Chemistry

**Scheme 1.**
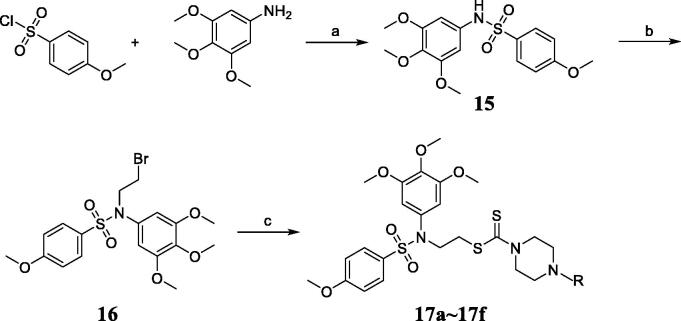
Synthesis of tertiary sulphonamide derivatives **17a**∼**17f**. Reagent and conditions: (a) Dichloromethane, KOH; (b) 1,2-Dibromoethane, acetone, K_2_CO_3_, 80 °C. (c) CS2, different piperazine analogues, acetone, Na_3_PO_4_.12H_2_O.

Tertiary sulphonamide derivatives **17a**∼**17f** were synthesised as shown in the Scheme 1. Sulphanilamide intermediate **15** was synthesised firstly in the high yield using 4-methoxybenzenesulfonyl chloride and 3,4,5-trimethoxyaniline. 1,2-Dibromoethane was reacted with the intermediate **15** to form compound **16** in the presence of potassium carbonate and acetone at 80 °C. Tertiary sulphonamide derivatives **17a**∼**17f** were obtained by the reaction of intermediate **15**, different piperazines and carbon disulphide in the presence of Na_3_PO_4_.12H_2_O.

### 3.2. Antiproliferative activity

Globally, liver cancer is one of the diseases leading to human death, and it is a serious threat for life health^12^. There are many factors to trigger liver cancer, including alcohol consumption, hepatitis B/C virus infection, toxic substances, and metabolic disorders^13^. Although many patients with liver cancer have been diagnosed and treated at an early stage, its recurrence rates and mortality rates are still very high^14^. Hence, it is of great clinical significance to develop novel bioactive agents for liver cancer therapy.

In order to discover the potent anti-liver cancer agents, all tertiary sulphonamide analogues were evaluated for their anticancer activity *in vitro* against liver cancer cell lines (SNU-423, HepG-2 and Bel-7402). Cell lines were treat with different tertiary sulphonamide derivatives **16** and **17a**∼**17f** for 48 h to obtain IC_50_ values. In this work, 5-fluorouracil as an well-known antitumor drug was selected as the control agent. The inhibitory activity results of tertiary sulphonamide derivatives **16** and **17a**∼**17f** were showed in Table 1.

**Table 1.** Antiproliferative activity of compounds **17a**∼**17f** against liver cancer cell lines for 48 h.

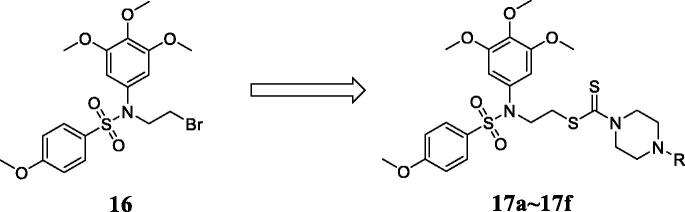				
Compound	R	IC_50_ (μM)		
		SNU-475	Bel-7402	HepG-2
**16**	–	> 20	> 20	> 20
**17a**	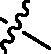	0.73 ± 0.16	0.32 ± 0.08	0.57 ± 0.07
**17b**	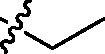	2.35 ± 0.70	1.37 ± 0.16	1.03 ± 0.28
**17c**	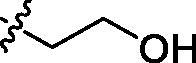	10.76 ± 1.01	7.09 ± 1.33	9.17 ± 2.18
**17d**	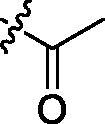	5.06 ± 0.68	1.58 ± 1.02	3.29 ± 0.84
**17e**	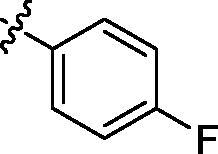	6.17 ± 1.05	9.18 ± 1.32	7.57 ± 0.96
**17f**	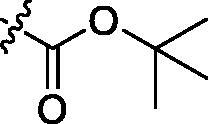	4.46 ± 1.38	2.32 ± 1.63	6.67 ± 1.25
5-Fluorouracil	**–**	26.47 ± 0.16	15.10 ± 1.28	12.17 ± 0.68

The replacement of bromine atom of compound **16** by the dithiocarbamate fragment of tertiary sulphonamide derivatives **17a**∼**17f** led to a significant improvement of inhibitory activity against liver cancer cell lines. Especially, tertiary sulphonamide derivatives **17a**∼**17f** displayed the better antiproliferative effects with IC_50_ values from 0.32 μM to 10.76 μM than 5-fluorouracil. These results demonstrated that the dithiocarbamate fragment might display synergistic effects for these tertiary sulphonamide derivatives against liver cancer cells.

Among all tertiary sulphonamide derivatives, compound **17a** showed the best antiproliferative activity with IC_50_ values of 0.73 μM, 0.32 μM, and 0.57 μM against SNU-423, Bel-7402, and HepG-2 cells. For tertiary sulphonamide derivatives **17a**∼**17f**, the substituent group attaching piperazine ring was significant for the antiproliferative activity exhibiting an over 8-fold activity increase against liver cancer HepG-2 cells (tertiary sulphonamide **17b *VS***. tertiary sulphonamide **17c**). Meanwhile, replacement of the t-butyloxycarboryl group of derivative **17f** with a acetyl group of derivative **17d** resulted in an decrease of the activity against HepG-2 and Bel-7402 cells. Tertiary sulphonamide derivatives **17a** and **17 b** were further explored for their cytotoxicity against L-02 cells (normal liver cell line). We discovered that derivatives **17a** and **17b** displayed no cytotoxic effects against L-02 cells (> 64 μM). These results demonstrated that tertiary sulphonamide derivatives in this work had the cytotoxic selectivity.

### 3.3. The inhibitory activity of tubulin polymerisation *in vitro*

**Figure 4.**
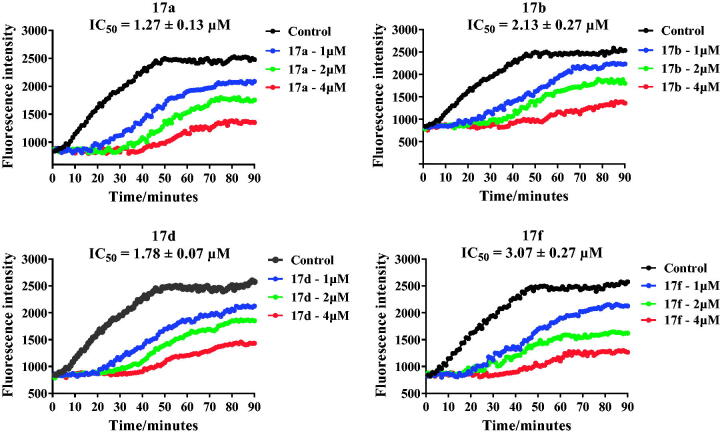
Tubulin polymerisation inhibitory activity of tertiary sulphonamide derivatives **17a**, **17b**, **17d** and **17f**.

Based on the inhibitory activity results of tertiary sulphonamide analogues against liver cancer cells, the more potent tertiary sulphonamides **17a**, **17b**, **17d** and **17f** were selected to evaluate their potential effects for tubulin polymerisation. The tubulin polymerisation inhibition activity of tertiary sulphonamide derivatives **17a**, **17b**, **17d** and **17f** was measured. As shown in Figure 4, the fluorescence intensity of tubulin was obviously decreased with the treatment of derivatives **17a**, **17b**, **17d** and **17f**. The IC_50_ value of derivatives **17a**, **17b**, **17d** and **17f** were 1.27 μM, 2.13 μM, 1.78 μM and 3.07 μM against tubulin. Replacing the methyl group (**17a**) by ethyl (**17b**), acetyl (**17d**) and tert-butoxycarbonyl (**17f**) caused a decrease of activity, indicating that substituents attaching the piperazine fragment displayed the important effects against tubulin. Results of their inhibitory activity investigated that these derivatives were novel tubulin polymerisation inhibitors.

### 3.4. Immunofluorescence of tertiary sulphonamide derivatives

**Figure 5.**
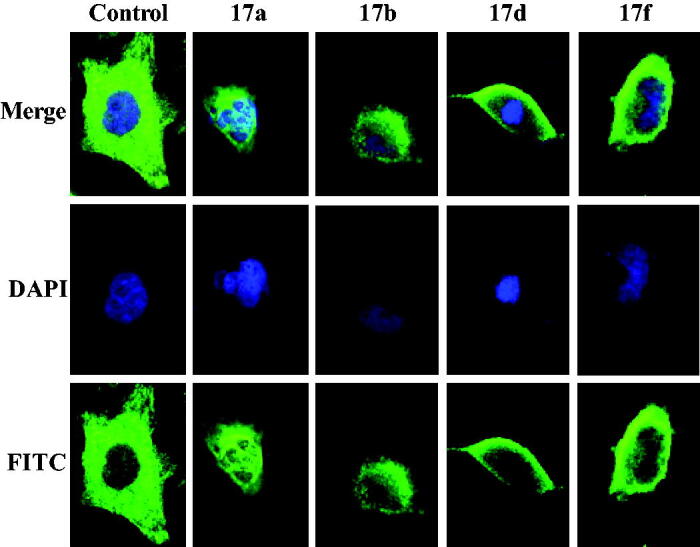
Tertiary sulphonamide derivatives showed the depolymerising effects on the interphase microtubule network.

To investigate the depolymerisation effects to microtubules, Bel-7402 cells were treated with tertiary sulphonamide derivatives **17a**, **17b**, **17d** and **17f** at 50 nM to perform immunofluorescence. As shown in Figure 5, tertiary sulphonamide derivative **17f** moderately depolymerised interphase microtubules whereas tertiary sulphonamide derivatives **17a** and **17b** produced a more pronounced depolymerisation effects. These immunofluorescence experiments also demonstrated that these tertiary sulphonamide derivatives were tubulin polymerisation inhibitors.

### 3.5. Docking analysis of tertiary sulphonamide derivative 17a targeting tubulin

**Figure 6.**
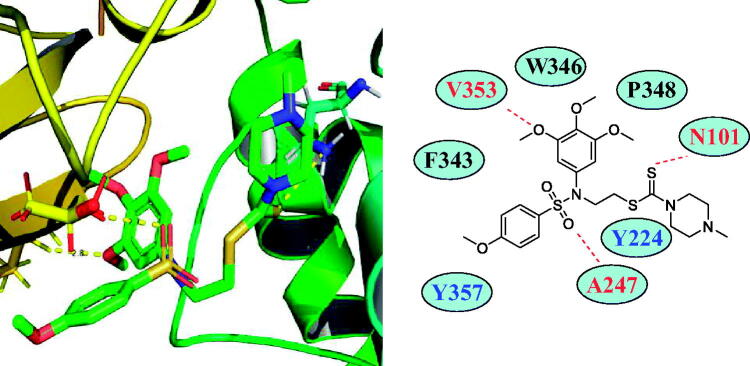
Molecular docking studies of tertiary sulphonamide derivative **17a** targeting tubulin (PDB code: 1SA0).

Based on its inhibitory effects against tubulin polymerisation, tertiary sulphonamide derivative **17a** was identified as a new tubulin polymerisation inhibitor. In this work, molecular docking studies between tertiary sulphonamide derivative **17a** and tubulin were explored. Autodock software was used to analyse the binding modes and the selected PDB code was 1SA0. From the docking results of Figure 6, the 3,4,5-trimethoxyphenyl fragment and tertiary sulphonamide moiety formed two hydrogen bonds with residues of V353 and A247. In addition, dithiocarbamate formed a hydrogen bond with N101. These results might demonstrate that derivatives **17a**∼**17f** were more potent than tertiary sulphonamide **16** without a dithiocarbamate fragment for the antiproliferative activity against liver cancer. Tertiary sulphonamide derivative **17a** also formed the hydrophobic effects with residues of W346, P348 and F343. As a reference ligand, colchicine was docked using the same PDB code. In the Supplementary Figure 1(S), colchicine (blue structure) was located into a different pocket compared with **17a** (red structure).

### 3.6. Tertiary sulphonamide derivatives inhibit LSD1 at the enzymatic level

**Figure 7.**
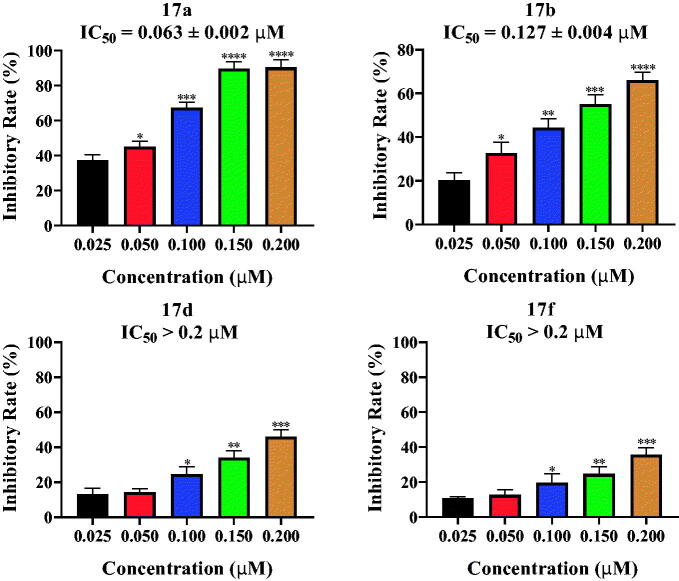
Tertiary sulphonamide derivatives inhibited LSD1 in a concentration dependent manner.

To determine the inhibitory effects of these tertiary sulphonamide derivative against LSD1, LSD1 enzymatic assay was performed according to reported references^15^^,^^16^. The enzymatic activity results of tertiary sulphonamide derivatives against LSD1 were shown in Figure 7. Because of their potent antiproliferative activity against liver cancer, tertiary sulphonamide derivatives **17a**, **17b**, **17d** and **17f** were selected to do this experiment. Among these four compounds, tertiary sulphonamide derivatives **17a** and **17b** exhibited the better inhibitory activity with IC_50_ values of 0.063 μM and 0.127 μM. However, tertiary sulphonamide derivatives **17d** and **17f** displayed the moderate inhibition effects against LSD1 with IC_50_ values of > 0.2 μM. The inhibitory rates of tertiary sulphonamide derivatives **17d** and **17f** at 0.2 μM were 46.0% and 35.7%, respectively. During the SAR studies, we found that substitutions on the piperazine ring was important for the inhibitory activity against LSD1 showing over 3-fold activity loss, when the methyl group was replaced with the acetyl group (**17d** *VS*. **17f**).

From these results, tertiary sulphonamide derivatives could concentration-dependently inhibit LSD1, indicating that they might be novel LSD1 inhibitors.

### 3.7. Tertiary sulphonamide derivative 17a inhibits LSD1 against liver cancer bel-7402 cells

**Figure 8.**
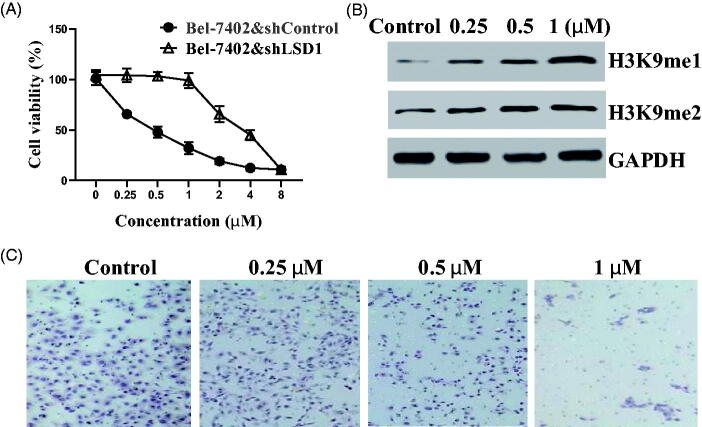
Tertiary sulphonamide derivative **17a** inhibits LSD1 against liver cancer Bel-7402 cells. (**A**) Antiproliferative effects of **17a** against Bel-7402&shLSD1 and Bel-7402&shControl. (**B**) Expression levels of H3K9me1 and H3K9me2 in Bel-7402 cells with the treatment of **17a**. (**C**) **17a** inhibited the migration against Bel-7402 cells.

Recent investigation has demonstrated that LSD1 is aberrantly overexpressed in different liver cancer cell lines^17^. LSD1 inhibition by small molecule inhibitors has been an effective strategy to suppress liver cancer^18^. Because of the best LSD1 inhibitory effects, **17a** was selected to perform the further investigation. LSD1 knock-down Bel-7402 cells (Bel-7402&shLSD1) and control cells (Bel-7402&shControl) were cultured and treated with **17a** according to similar methods^19^^,^^20^. As shown in Figure 8(A), tertiary sulphonamide derivative **17a** potently inhibited the cell viability of Bel-7402&shControl cells. In contrast, compound **17a** moderately inhibited Bel-7402&shLSD1 cells, about 8 ∼ 9 fold less potent against Bel-7402&shControl cells. These results suggested that the antiproliferative effects of **17a** against liver cancer Bel-7402 cells were dependent on the LSD1 inhibition.

LSD1 could specifically demethylate mono- and dimethyl-lysine on histone H3 H3K9me1/2), leading to the transcriptional repression or activation of target genes ^21^. Thus, the expression levels of H3K9me1/2 in liver cancer Bel-7402 cells were explored with the treatment of **17a**. From the experimental results in Figure 8(B), tertiary sulphonamide derivative **17a** increased the expression levels of H3K9me1 and H3K9me2, indicating that **17a** is a cellular active LSD1 inhibitor in liver cancer Bel-7402 cells. Recent studies demonstrated that the inhibition of LSD1 repressed the migration of liver cancer cells^22^. The migration effects of tertiary sulphonamide derivative **17a** were also investigated. As shown in Figure 8(C), tertiary sulphonamide derivative **17a** significantly inhibited the migration against liver cancer Bel-7402 cells.

### 3.8. Docking analysis of tertiary sulphonamide derivative 17a targeting LSD1

**Figure 9.**
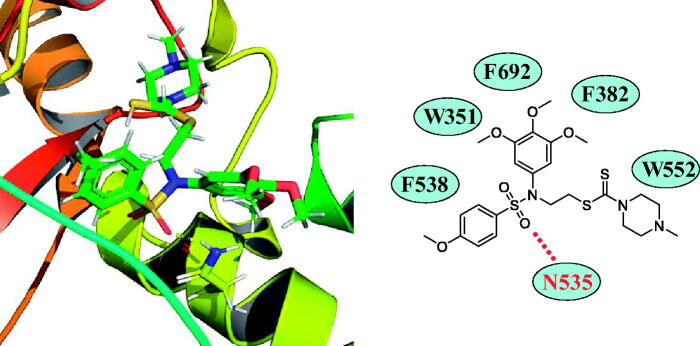
Molecular docking studies of **17a** targeting LSD1 (PDB code: 2H94).

Analysis of the binding modes between **17a** and LSD1 was performed via the autodock software. The selected PDB code of LSD1 was 2H94 and its resolution was 2.90 Å. The binding modes demonstrated that the oxygen atom of tertiary sulphonamide group could form a hydrogen bond with N535 (Figure 9). Furthermore, F538, W351, F692, F382 and W552 formed the hydrophobic pocket that surrounds the 4-methoxyphenyl ring and 3,4,5-trimethoxyphenyl moiety of tertiary sulphonamide derivative **17a**. These results further supported our findings about the LSD1 inhibition of tertiary sulphonamide derivative **17a**. As a reference ligand, FAD was docked using the same PDB code (2H94). In the Supplementary Figure 2(S), FAD (blue structure) was located into a different pocket compared with **17a** (red structure).

### 3.9. *In vivo* antitumour study of tertiary sulphonamide derivative 17a

**Figure 10.**
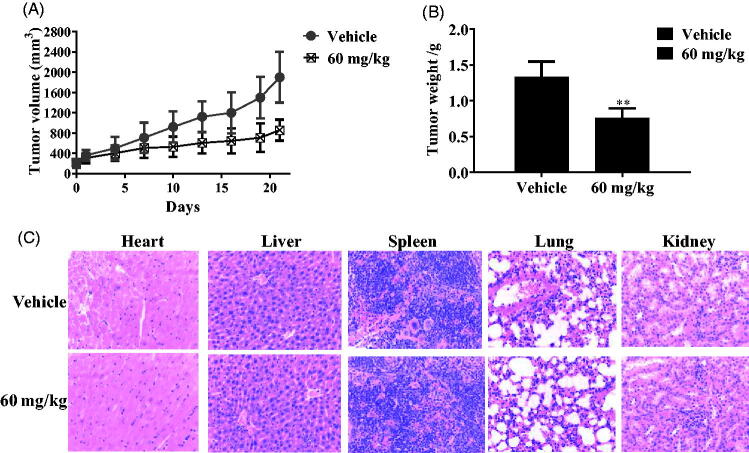
Antitumor effects of tertiary sulphonamide derivative **17a**. (**A**) Tumour volume of treated mice. (**B**) Tumour weight of treated mice. (**C**) Haematoxylin & eosin staining of heart, liver, spleen, lung and kidney.

Based on the potent anticancer activity *in vitro* of **17a** against Bel-7402 cells, its tumour growth effects *in vivo* was next examined using a xenograft model. Xenograft tumours were produced by the subcutaneous implantation of liver cancer Bel-7402 cells. With the treatment of tertiary sulphonamide derivative **17a** by the intragastric administration, tumour weight of the mice and their tumour volume were measured (Figure 10(A,B)). The treatment at a dose of 60 mg/kg of **17a** inhibited the tumour growth and reduced the tumour weight by 42.97%. As shown in Figure 10(C), there were no apparent toxicity of organs (heart, liver, spleen, lung and kidney) during the treatment. These data demonstrated that **17a** was a novel anticancer agent without the obvious global toxicity.

## 4. Conclusion

In conclusion, we have synthesised a new series of tertiary sulphonamide derivatives and evaluated for their anti-liver cancer activity *in vitro* and *in vivo*. Some of tertiary sulphonamides, especially compound **17a**, exhibit the antiproliferative activity with IC_50_ values of 0.73 μM, 0.32 μM, and 0.57 μM against SNU-423, Bel-7402, and HepG-2 cells, respectively. Tertiary sulphonamide derivative **17a** could also effectively inhibited the tumour growth *in vivo*. Further mechanisms investigated that tertiary sulphonamide derivative **17a** inhibited tubulin polymerisation and depolymerised interphase microtubules. It suppressed LSD1 in a enzymatic level and cellular level by the accumulation of H3K9me1 and H3K9me2. It is the first time to reveal that this tertiary sulphonamide derivative might be a dual inhibitor of LSD1 and tubulin polymerisation. Our findings indicated that this tertiary sulphonamide scaffold could be a novel antitumor skeleton to design more potent molecules for liver cancer therapy.

## Supplementary Material

Supplemental Material
