# Supplementary material for: Tertiary sulphonamide derivatives as dual acting small molecules that inhibit LSD1 and suppress tubulin polymerisation against liver cancer
Source: J Enzyme Inhib Med Chem. 2021 Jul 19;36(1):1563–72. doi: 10.1080/14756366.2021.1917564 (PMC8291071; doi:10.1080/14756366.2021.1917564)
Supplement: Supplemental Material [file IENZ_A_1917564_SM6677.pdf]

Novel sulfanilamide derivatives as dual acting small molecules that inhibit LSD1 and suppress tubulin polymerization against liver cancer

Lijuan Ding[a1], Feng Wei[a1], Nanya Wang[a], Yue Sun[a], Qiang Wang[a], Xia Fan[a], Ling Qi[*b] and Shudong Wang[*a]

[a]The First Hospital of Jilin University, Changchun, 130021, China.

[b]The Sixth Affiliated Hospital of Guangzhou Medical University, Qingyuan People's Hospital, Qingyuan, 511518, China.

[*]Corresponding author: Ling Qi (qiling1718@gzhmu.edu.cn); Shudong Wang (shudong_wang@jlu.edu.cn).

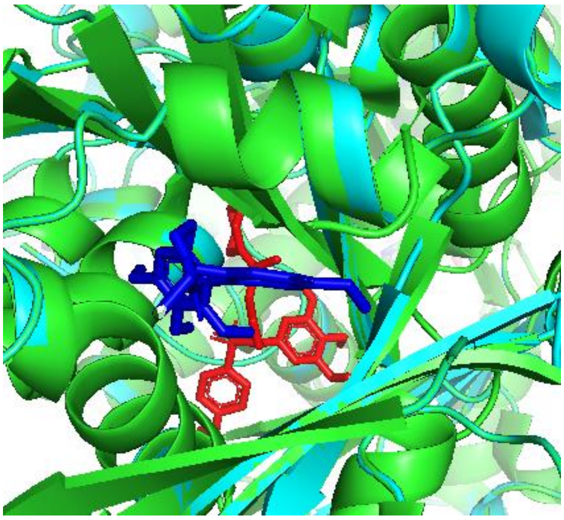

Figure 1S. Docking pocket between colchicine and tertiary sulfonamide derivative **17a**. (colchicine: blue structure; **17a**: red structure).

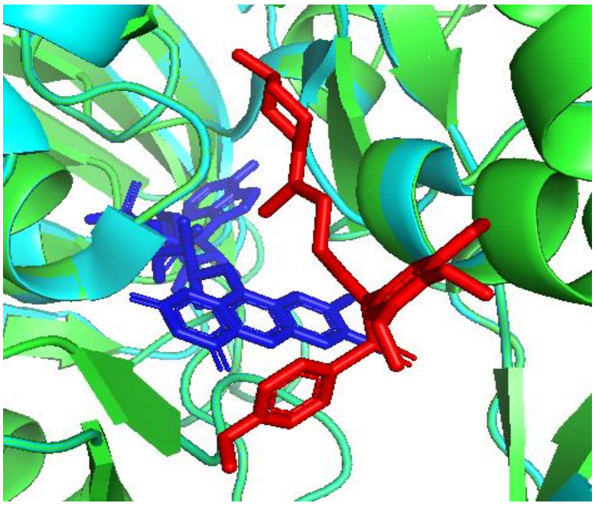

Figure 2S. Docking pocket between FAD and tertiary sulfonamide derivative **17a**. (FAD: blue structure; **17a**: red structure).

4-Methoxy-N-(3,4,5-trimethoxyphenyl)benzenesulfonamide (15)

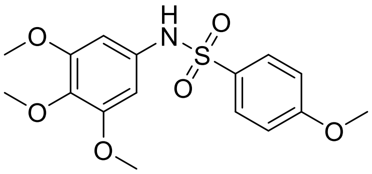

Yield: 84%, white solid, m.p.:114~116 °C. ^{1}H NMR (400 MHz, DMSO-d_6) δ 9.97 (s, 1H), 7.78 – 7.65 (m, 2H), 7.16 – 7.00 (m, 2H), 6.38 (s, 2H), 3.80 (s, 3H), 3.65 (s, 6H), 3.56 (s, 3H). ^{13}C NMR (100 MHz, DMSO-d_6) δ 162.43, 152.91, 133.99, 133.84, 131.03, 129.01, 114.32, 97.72, 60.01, 55.70, 55.59. HRMS (m/z) calcd. $C_{16}H_{20}NO_6S$, $[M+H]^+$ m/z: 354.1016, found: 354.1011.

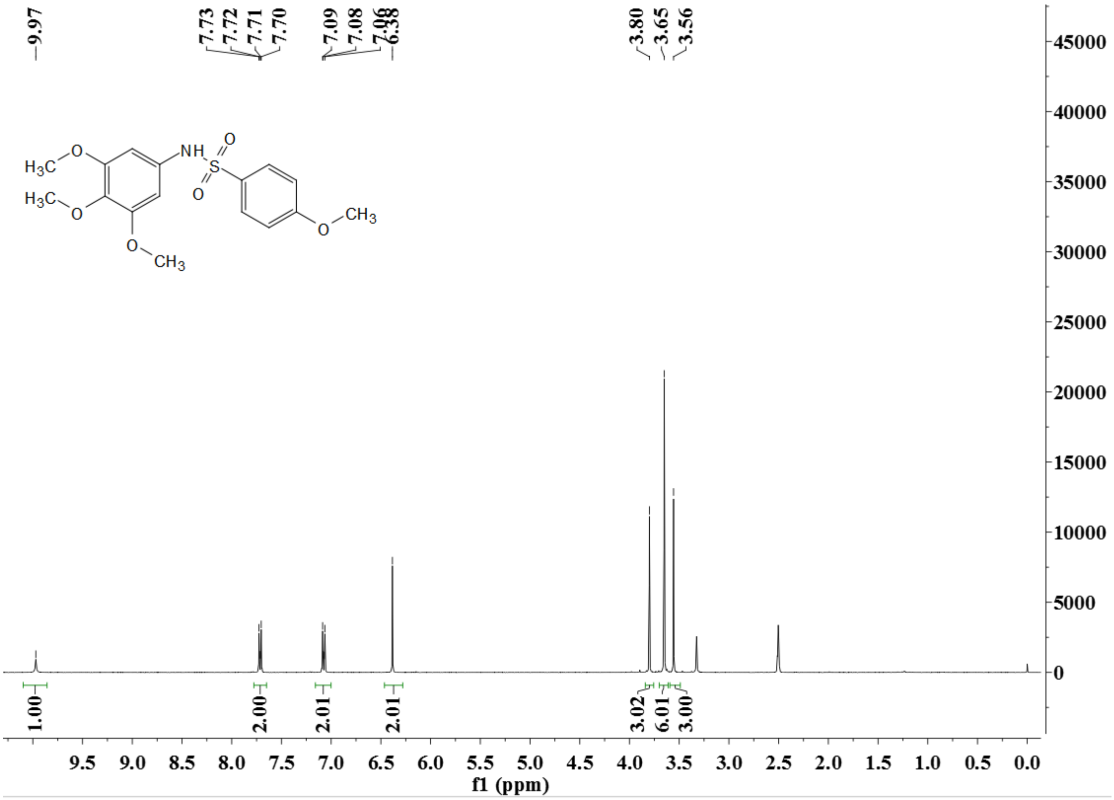

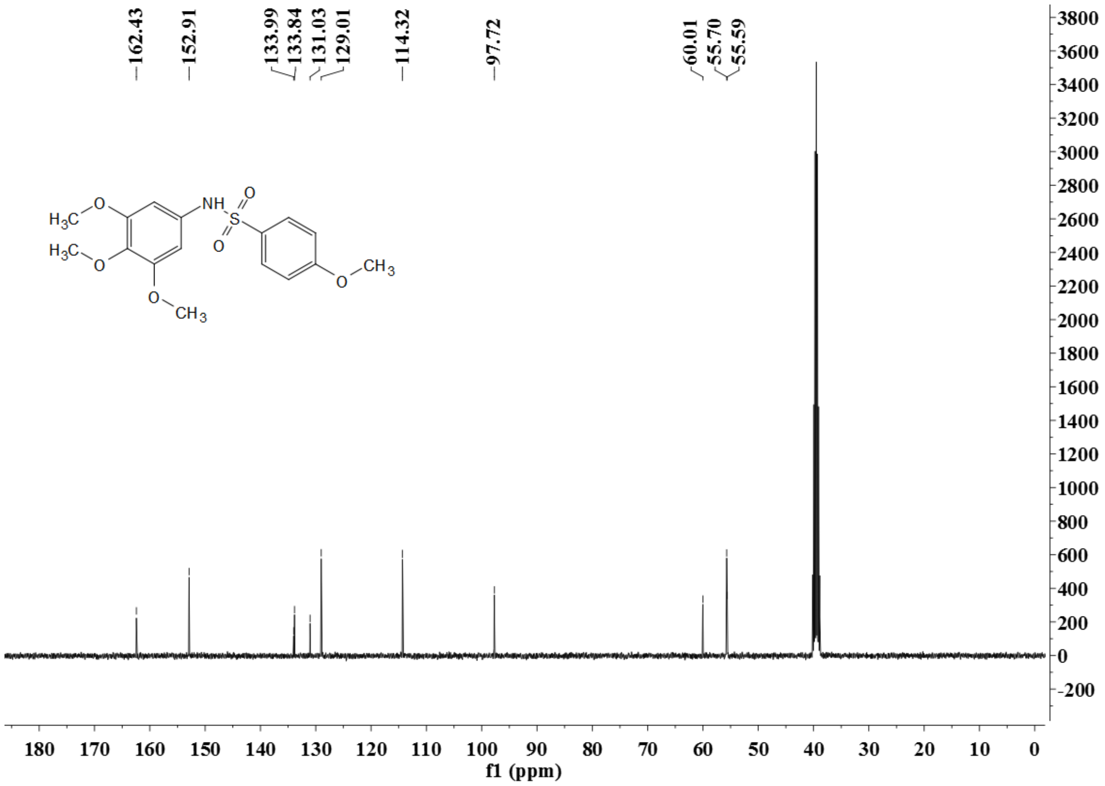

N-(2-bromoethyl)-4-methoxy-N-(3,4,5-trimethoxyphenyl)benzenesulfonamide (16)

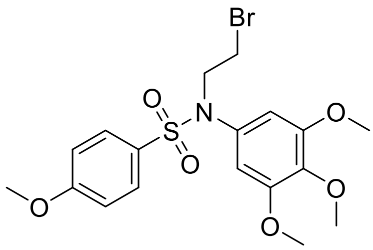

Yield: 90%, white solid, m.p.:116~118 °C. ^{1}H NMR (400 MHz, $CDCl_3$) δ 7.60 – 7.43 (m, 2H), 6.95 – 6.80 (m, 2H), 6.18 (s, 2H), 3.78 (dd, *J* = 12.2, 6.5 Hz, 8H), 3.65 (s, 6H), 3.34 (t, *J* = 7.4 Hz, 2H). ^{13}C NMR (100 MHz, $CDCl_3$) δ 162.21, 152.27, 137.24, 133.46, 129.03, 128.75, 112.96, 105.60, 59.90, 55.18, 54.67, 51.75, 27.96. HRMS (m/z) calcd. $C_{18}H_{23}BrNO_6S$, $[M+H]^+$ m/z: 460.0434, found: 460.0429.

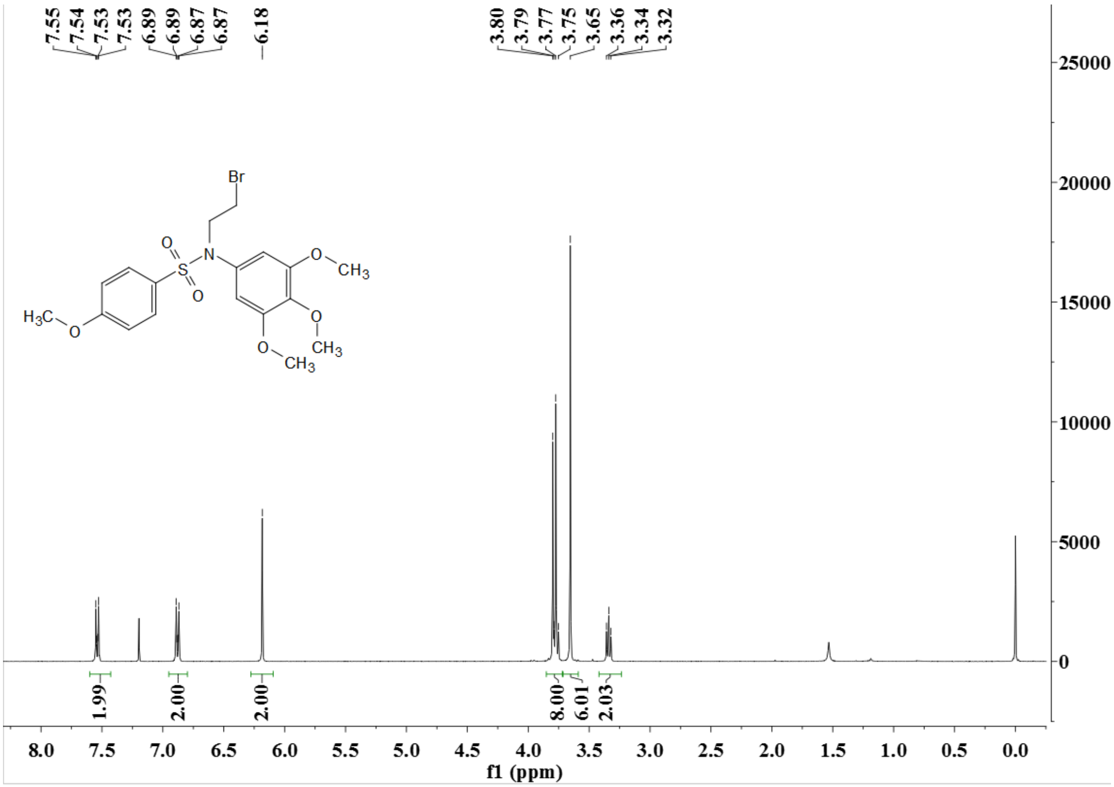

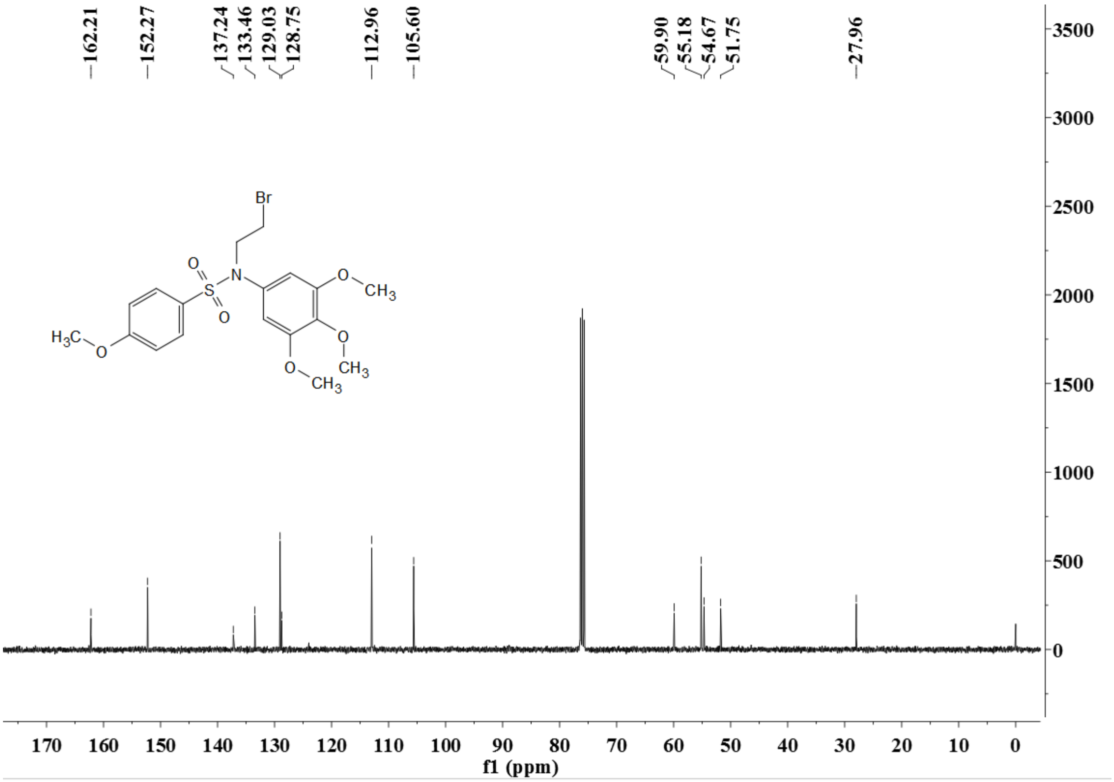

2-((4-Methoxy-N-(3,4,5-trimethoxyphenyl)phenyl)sulfonamido)ethyl-4-methylpiperazine-1-carbodithioate (17a)

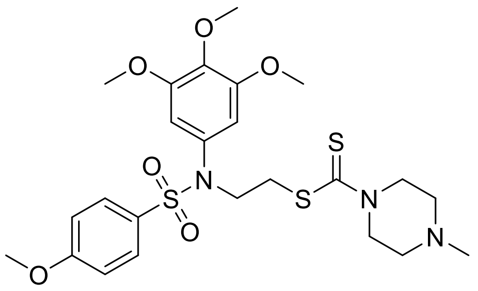

Yield: 67%, white solid, m.p.:142~144 °C. ^{1}H NMR (400 MHz, $CDCl_3$) δ 7.51 (d, J = 8.8 Hz, 2H), 6.86 (d, J = 8.9 Hz, 2H), 6.24 (s, 2H), 4.23 (s, 2H), 3.85 (s, 2H), 3.78 (d, J = 3.5 Hz, 6H), 3.76 – 3.70 (m, 2H), 3.67 (s, 6H), 3.46 – 3.23 (m, 2H), 2.50 – 2.31 (m, 4H), 2.25 (s, 3H). ^{13}C NMR (100 MHz, $CDCl_3$) δ 194.81, 162.05, 152.03, 136.91, 133.38, 129.01, 128.68, 112.88, 105.42, 59.91, 55.18, 54.64, 53.33, 48.62, 44.58, 33.99. HRMS (m/z) calcd. $C_{24}H_{34}N_3O_6S_3$, $[M+H]^+$ m/z: 556.1616, found: 556.1610.

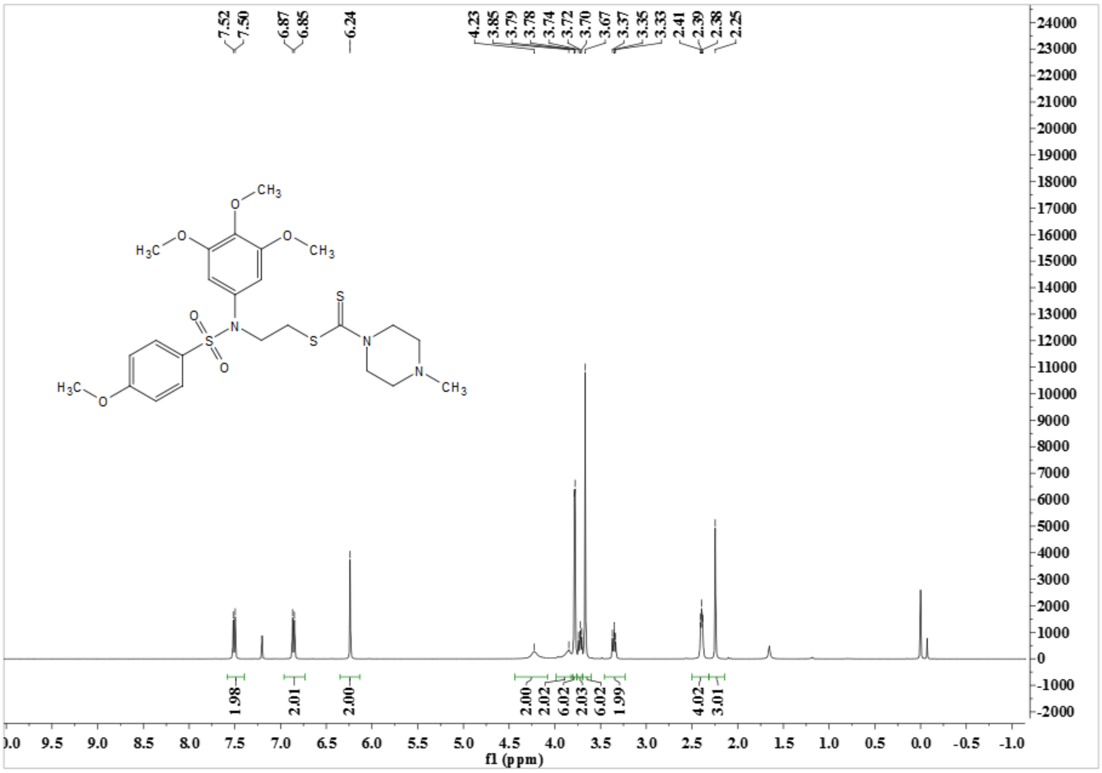

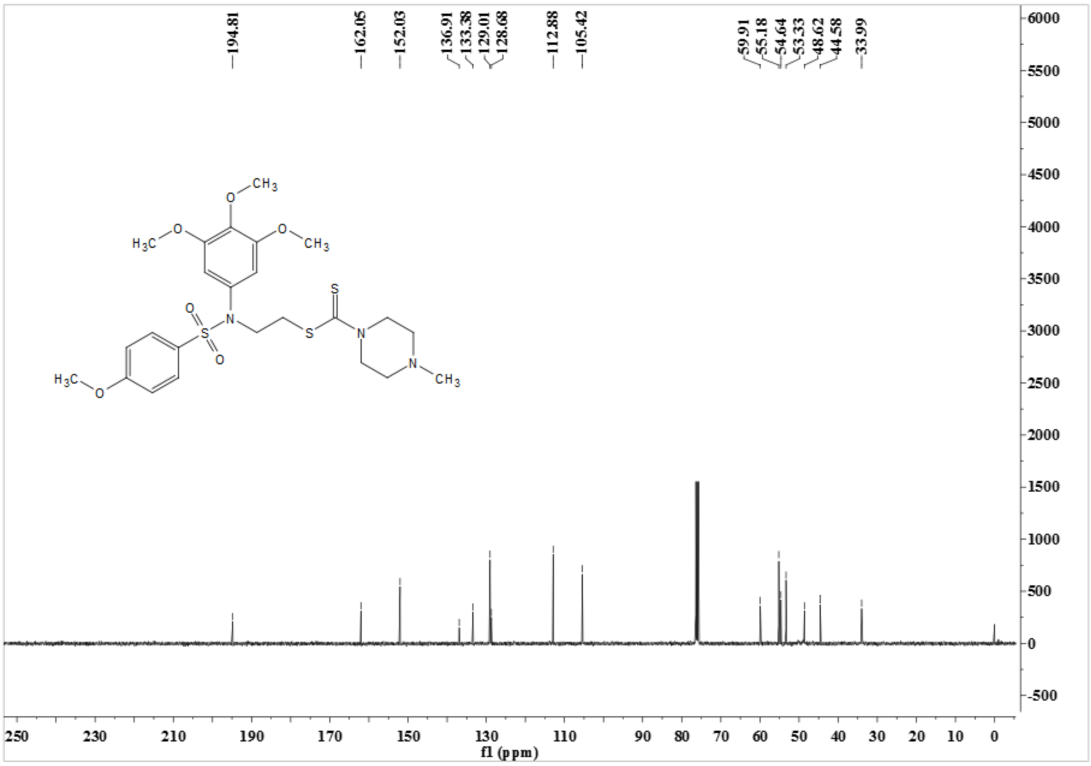

2-((4-Methoxy-N-(3,4,5-trimethoxyphenyl)phenyl)sulfonamido)ethyl-4-ethylpiperazine-1-carbodithioate (17b)

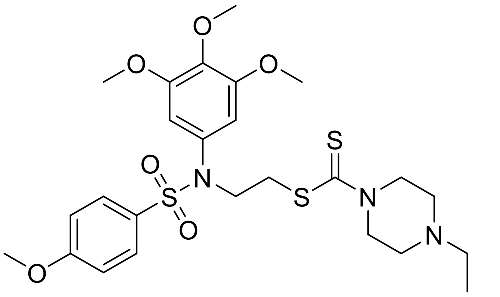

Yield: 86%, white solid, m.p.:150~152 ^{o}C. ^{1}H NMR (400 MHz, $CDCl_3$) δ 7.51 (d, *J* = 8.9 Hz, 2H), 6.97 – 6.75 (m, 2H), 6.24 (s, 2H), 4.23 (s, 2H), 3.85 (s, 2H), 3.78 (d, *J* = 3.5 Hz, 6H), 3.75 – 3.70 (m, 2H), 3.67 (s, 6H), 3.43 – 3.28 (m, 2H), 2.57 – 2.25 (m, 6H), 1.03 (t, *J* = 7.2 Hz, 3H). ^{13}C NMR (100 MHz, $CDCl_3$) δ 194.59, 162.04, 152.02, 136.90, 133.37, 129.01, 128.68, 112.88, 105.41, 59.91, 55.18, 54.63, 51.10, 50.85, 48.63, 33.95, 10.95. HRMS (m/z) calcd. $C_{25}H_{36}N_3O_6S_3$, $[M+H]^+$ m/z: 570.1769, found: 570.1766.

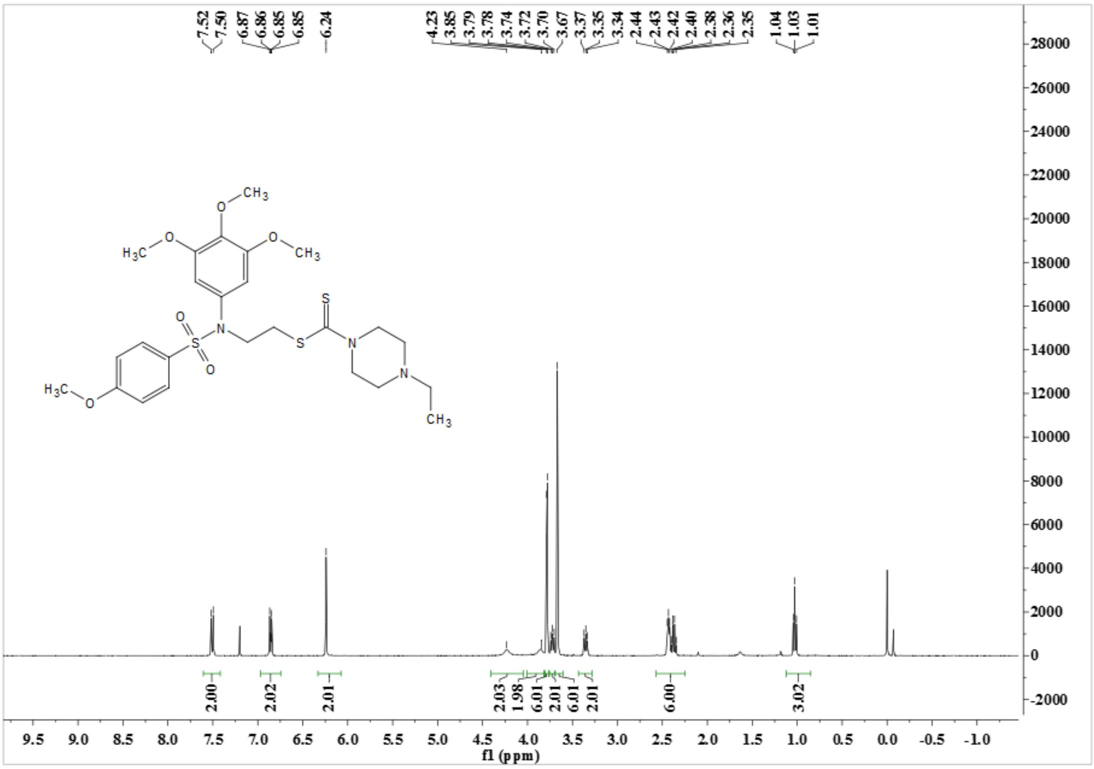

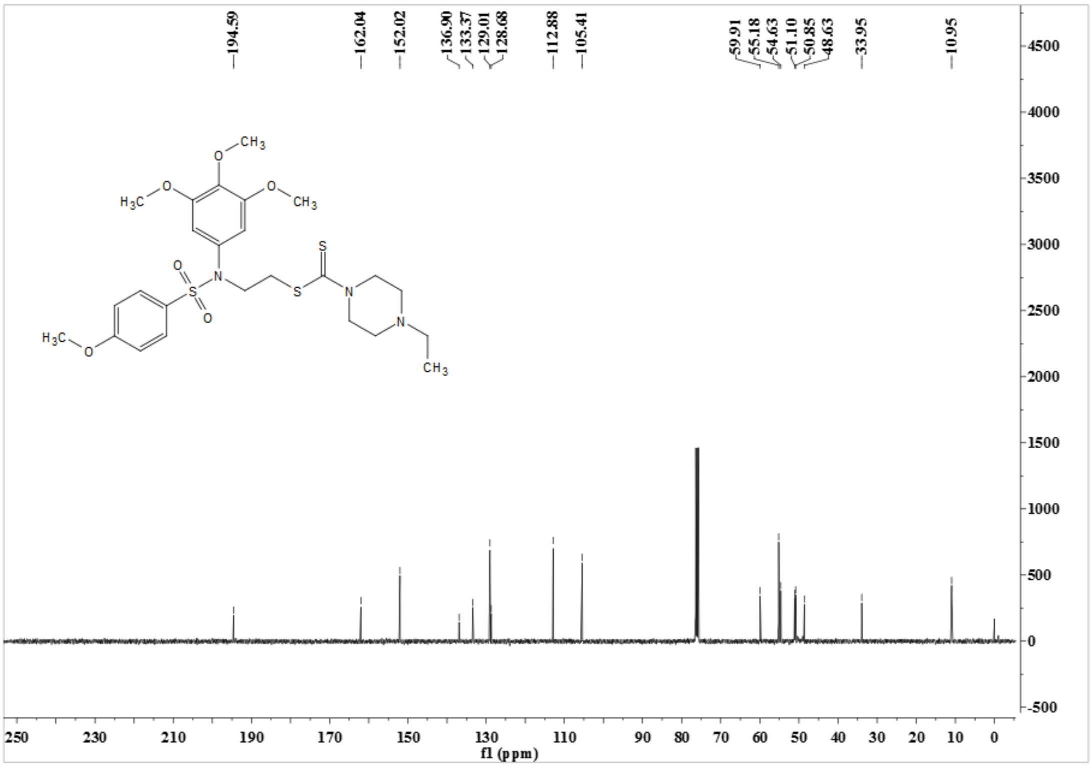

2-((4-Methoxy-N-(3,4,5-trimethoxyphenyl)phenyl)sulfonamido)ethyl-4-(2-hydroxyethyl)piperazine-1-carbodithioate (17c)

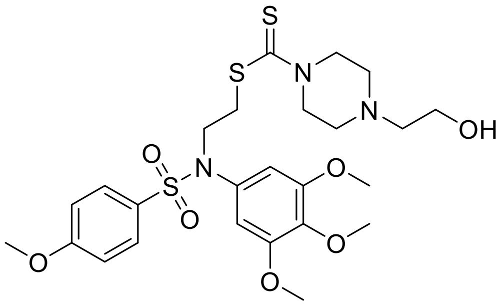

Yield: 90%, white solid, m.p.:146~148 °C. ¹H NMR (400 MHz, DMSO-d_6) δ 7.57 (d, *J* = 8.8 Hz, 2H), 7.12 (d, *J* = 8.9 Hz, 2H), 6.29 (s, 2H), 4.47 (t, *J* = 5.3 Hz, 1H), 4.18 (s, 2H), 3.84 (s, 5H), 3.75 (t, *J* = 6.8 Hz, 2H), 3.66 (s, 3H), 3.64 (s, 6H), 3.51 (q, *J* = 5.9 Hz, 2H), 3.29 (t, *J* = 6.9 Hz, 2H), 2.48 (d, *J* = 5.0 Hz, 4H), 2.42 (s, 2H). ¹³C NMR (100 MHz, DMSO-d_6) δ 194.00, 162.79, 152.57, 137.25, 134.06, 129.79, 129.15, 114.31, 106.49, 60.06, 59.44, 58.48, 55.87, 55.74, 52.47, 49.07, 34.55. HRMS (m/z) calcd. $C_{25}H_{36}N_3O_7S_3$, $[M+H]^+$ m/z: 586.1719, found: 586.1715.

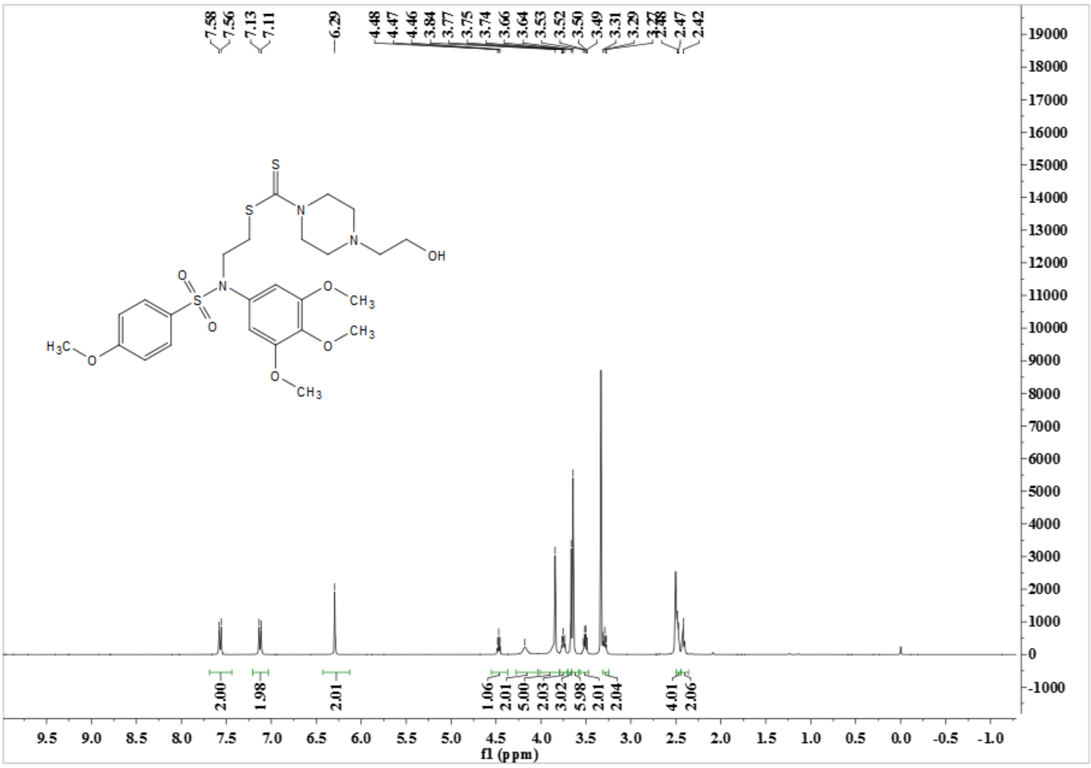

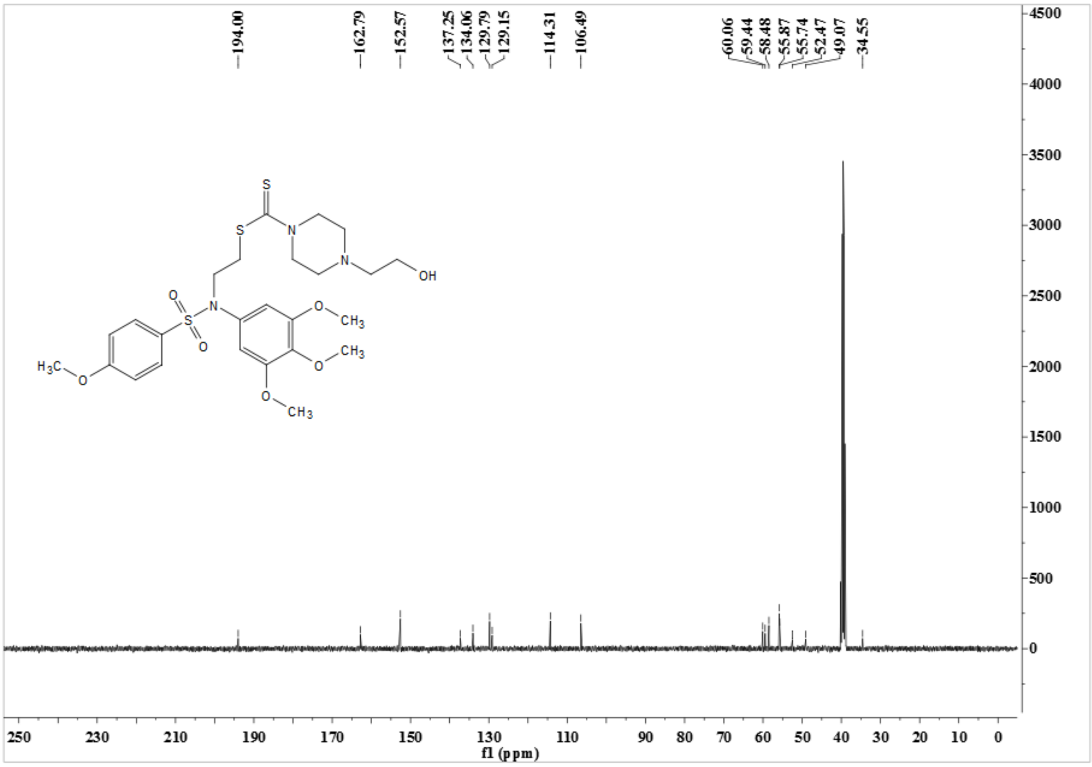

2-((4-Methoxy-N-(3,4,5-trimethoxyphenyl)phenyl)sulfonamido)ethyl-4-acetylpiperazine-1-carbodithioate (17d)

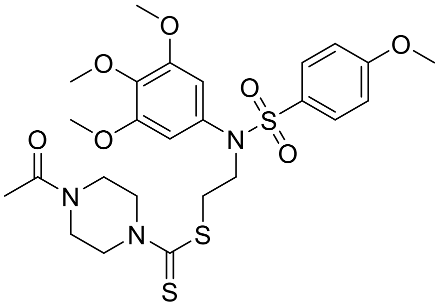

Yield: 90%, white solid, m.p.:152~154 ^{o}C. ^{1}H NMR (400 MHz, $CDCl_3$) δ 7.50 (d, *J* = 8.9 Hz, 2H), 6.86 (d, *J* = 8.9 Hz, 2H), 6.22 (s, 2H), 3.99 (dd, *J* = 70.5, 66.9 Hz, 4H), 3.78 (d, *J* = 3.6 Hz, 6H), 3.73 (t, *J* = 7.0 Hz, 2H), 3.66 (d, *J* = 8.5 Hz, 8H), 3.57 – 3.47 (m, 2H), 3.37 (t, *J* = 7.0 Hz, 2H), 2.06 (s, 3H). ^{13}C NMR (100 MHz, $CDCl_3$) δ 168.32, 162.09, 152.09, 136.98, 133.35, 128.99, 128.67, 112.91, 105.45, 59.92, 55.20, 54.66, 48.50, 44.14, 39.54, 34.12, 20.33. HRMS (m/z) calcd. $C_{25}H_{34}N_3O_7S_3$, $[M+H]^+$ m/z: 584.1565, found: 584.1559.

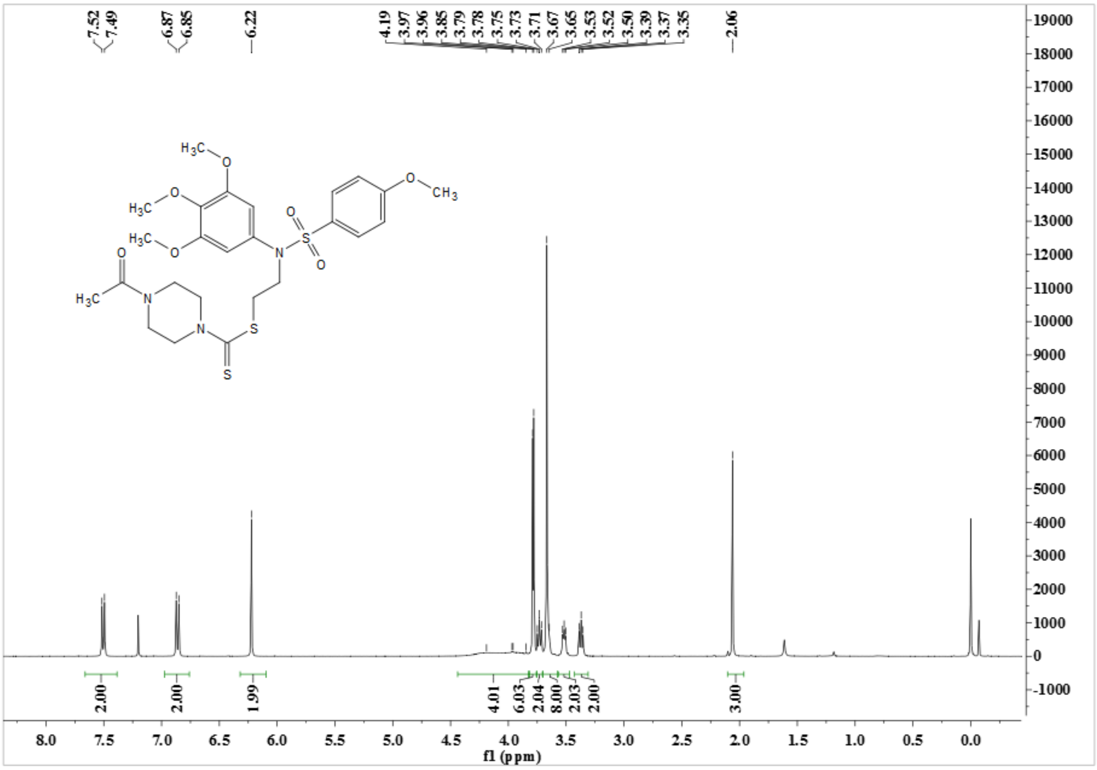

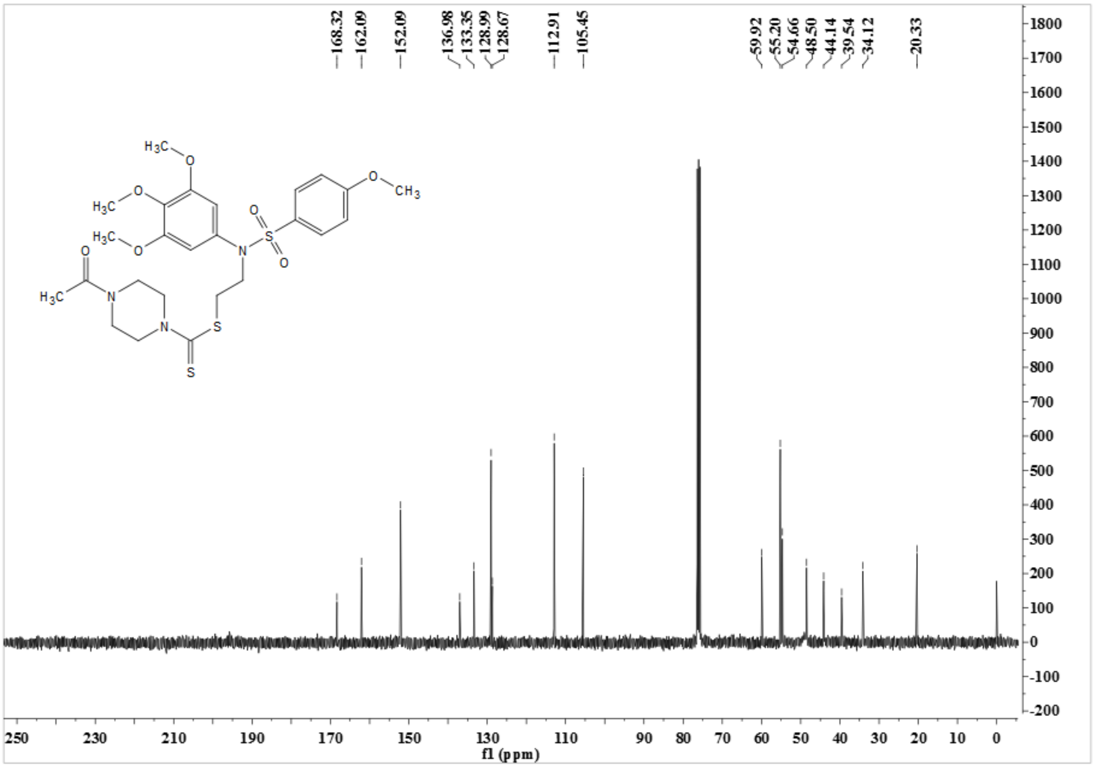

2-((4-Methoxy-N-(3,4,5-trimethoxyphenyl)phenyl)sulfonamido)ethyl-4-(4-fluorophenyl)piperazine-1-carbodithioate (17e)

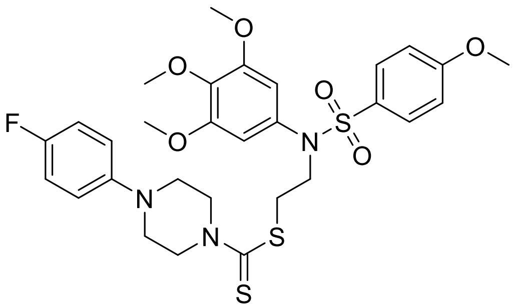

Yield: 72%, white solid, m.p.:166~168 ^{o}C. ^{1}H NMR (400 MHz, $CDCl_3$) δ 7.67 – 7.37 (m, 2H), 7.06 – 6.63 (m, 6H), 6.24 (s, 2H), 4.36 (s, 2H), 4.02 (s, 2H), 3.78 (s, 6H), 3.77 – 3.71 (m, 2H), 3.67 (s, 6H), 3.45 – 3.30 (m, 2H), 3.22 – 3.00 (m, 4H). ^{13}C NMR (100 MHz, $CDCl_3$) δ 195.14, 162.07, 152.08, 145.95, 133.38, 129.01, 117.51, 117.46, 117.38, 114.92, 114.69, 112.90, 105.50, 59.92, 55.21, 54.64, 48.86, 48.62, 34.08. HRMS (m/z) calcd. $C_{29}H_{35}FN_3O_6S_3$, [M+H] $^{+}$ m/z: 636.1678, found: 636.1672.

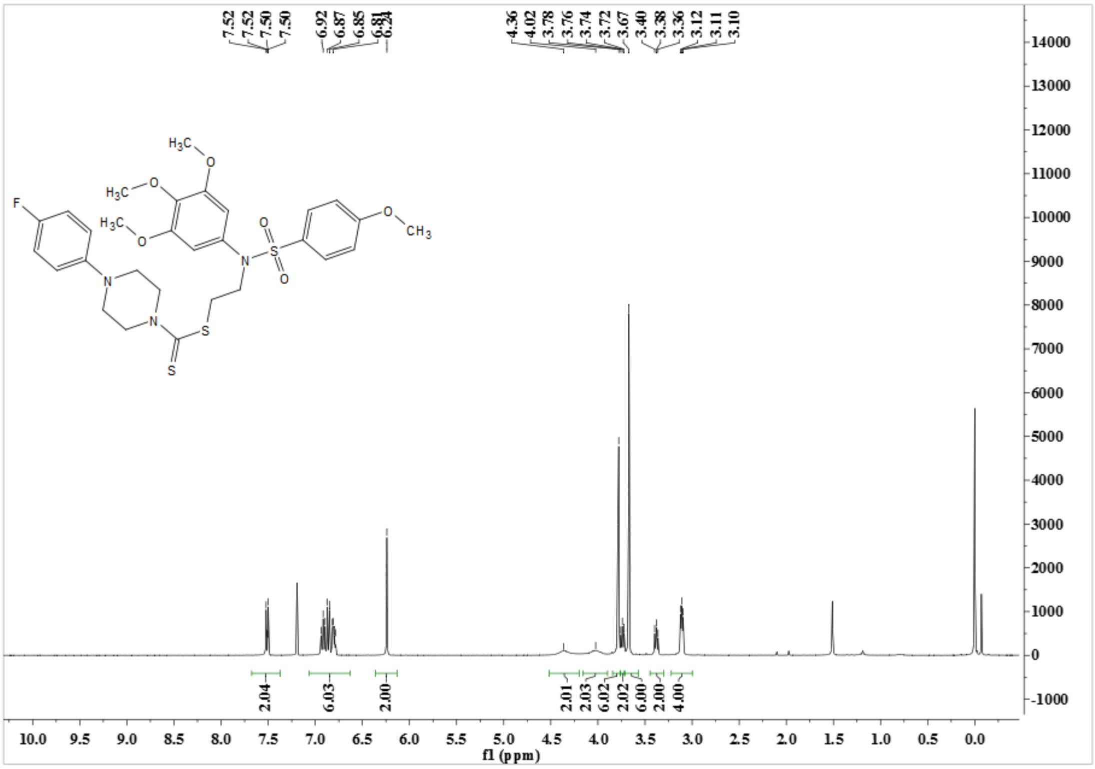

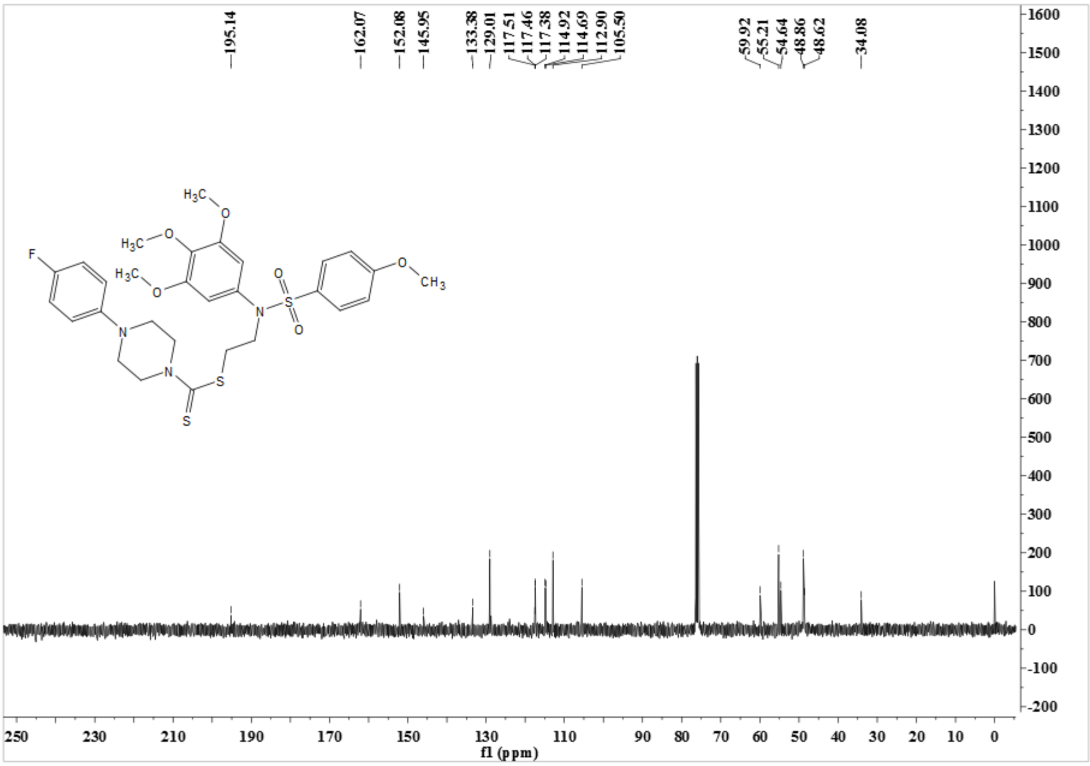

Tert-butyl-4-(((2-((4-methoxy-N-(3,4,5-trimethoxyphenyl)phenyl)sulfonamido)ethyl)thio)carbonothioyl)piperazine-1-carboxylate (17f)

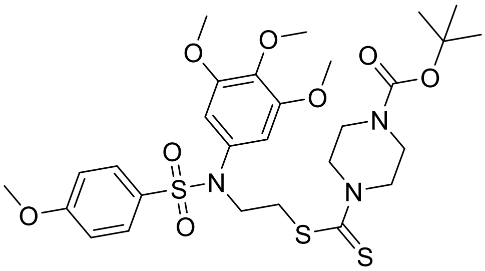

Yield: 52%, white solid, m.p.:162~164 ^{o}C. ^{1}H NMR (400 MHz, $CDCl_3$) δ 7.51 (d, *J* = 8.9 Hz, 2H), 6.86 (d, *J* = 8.9 Hz, 2H), 6.23 (s, 2H), 4.34 – 4.02 (m, 2H), 3.89 (dd, *J* = 54.5, 9.9 Hz, 2H), 3.78 (d, *J* = 3.6 Hz, 6H), 3.76 – 3.70 (m, 2H), 3.67 (s, 6H), 3.51 – 3.41 (m, 4H), 3.41 – 3.32 (m, 2H), 1.40 (s, 9H). ^{13}C NMR (100 MHz, $CDCl_3$) δ 195.42, 162.08, 153.42, 152.08, 137.01, 133.37, 129.01, 128.74, 112.90, 105.49, 79.64, 59.91, 55.21, 54.64, 48.57, 34.06, 27.35. HRMS (m/z) calcd. $C_{28}H_{40}N_3O_8S_3$, [M+H] $^{+}$ m/z: 642.1985, found: 642.1978.

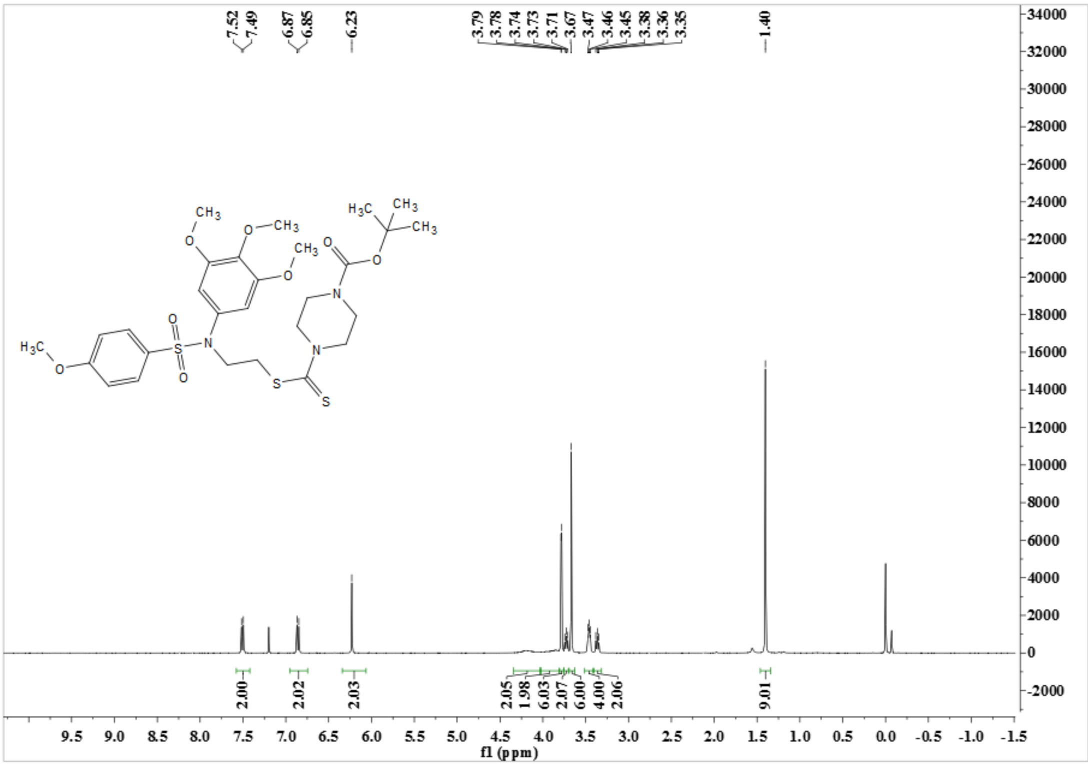

7.52
7.49
6.87
6.85
6.23
3.79
3.78
3.74
3.73
3.71
3.67
3.47
3.46
3.45
3.38
3.36
3.35
1.40
2.00
2.02
2.03
2.05
1.98
6.03
2.07
6.00
4.00
2.06
9.01
f1 (ppm)

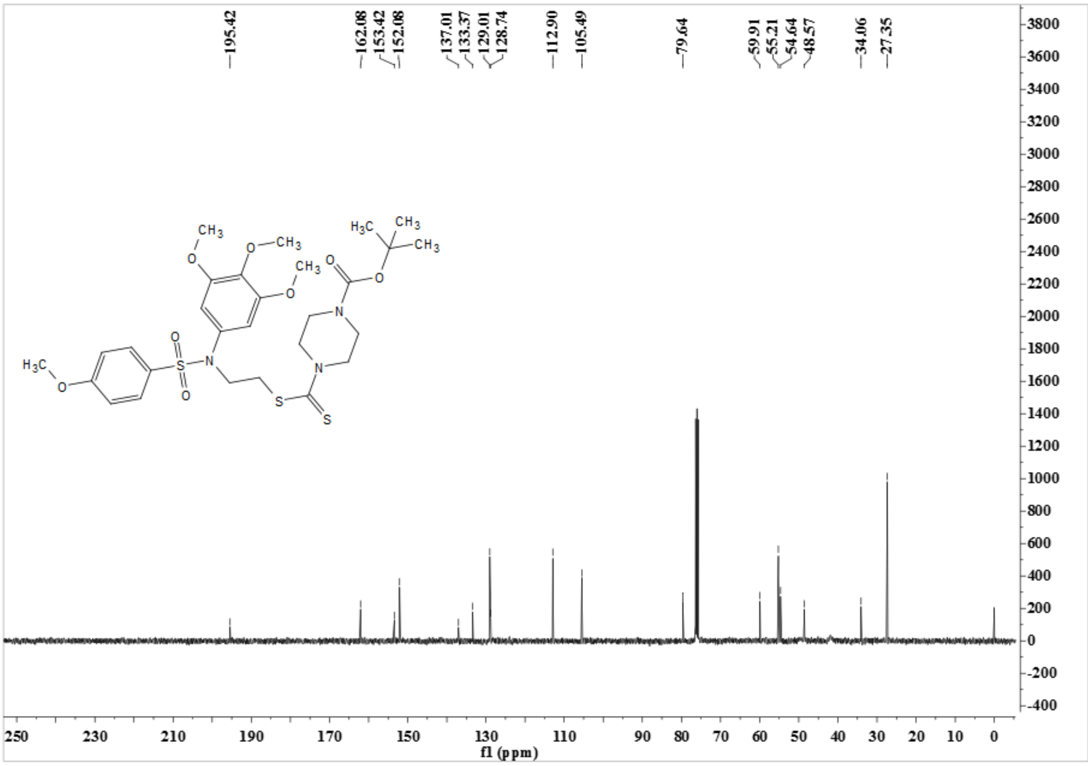

195.42
162.08
153.42
152.08
137.01
133.37
129.01
128.74
112.90
105.49
79.64
59.91
55.21
54.64
48.57
34.06
27.35
f1 (ppm)
